# Diabetic Retinopathy Prevalence and Incidence in Zimbabwe: The Feasibility of Digital Fundoscopy Screening

**DOI:** 10.1155/jdr/8048762

**Published:** 2026-01-26

**Authors:** Alvern N. Mutengerere, Adrian T. M. Musengi, Martina Kawome, Kudakwashe Madzeke, Manya Mirchandani, Alvin Ng, Laura E. Ruckstuhl

**Affiliations:** ^1^ SolidarMed, Masvingo, Zimbabwe; ^2^ Sally Mugabe Central Hospital, Ministry of Health and Child Care, Harare, Zimbabwe, mohcc.gov.zw; ^3^ Costello Medical, London, UK; ^4^ Costello Medical, Singapore, Singapore; ^5^ SolidarMed, Lucerne, Switzerland

**Keywords:** diabetes and endocrinology, diabetic retinopathy, preventive health services

## Abstract

Diabetic retinopathy (DR) is the leading cause of blindness in the working‐age population, yet many underserved communities lack access to screening programmes that would facilitate earlier diagnosis and access to treatment. This study is aimed at evaluating the feasibility of embedding digital fundoscopy (DF) for routine screening of patients with diabetes mellitus (DM) in Masvingo, Zimbabwe; assessing the prevalence and progression of DR among this previously unstudied population; and determining the baseline variables associated with DR and its progression. An observational study was conducted at Masvingo Provincial Hospital. Eligible participants were aged ≥ 18 and routinely attended the clinic. Participants (*N* = 202) were assessed for the presence and severity of DR using DF at baseline and again at 1 year. Images were sent to a remote ophthalmologist for diagnosis. Logistic regression was used to investigate the association of the participants′ demographics and medical history with DR. At baseline, 84 (41.6%) participants were diagnosed with DR. Among participants without DR at baseline, eight had DR at Year 1, translating to an annual incidence of 6.8%. Higher levels of haemoglobin A1c (HbA1c) were associated with increased odds of DR at baseline (*p* = 0.03) compared with normal HbA1c. The mean turnaround between image capture and clinical report availability at baseline (36.16 days) and at Year 1 (18.89 days) was aligned with global guidelines. High DR rates in Masvingo provide compelling evidence of the need for increased healthcare resources for DR screening in underserved settings; our study demonstrates the feasibility of embedding DF into standard practice for this purpose. Patients with poorly managed DM, as indicated by elevated HbA1c, should be prioritised for DF screening programmes and monitoring to facilitate early diagnosis and prevent avoidable blindness. Further investigation is needed into factors associated with DR progression.

## 1. Introduction

Diabetic retinopathy (DR), a progressive disease of the retinal microvasculature, is one of the most common complications of diabetes mellitus (DM), estimated to affect 22.3% of patients with DM worldwide [1, 2]. The prevalence of DR is particularly high in Africa, where 35.9% of diabetic patients were reported to have DR in 2020—a figure projected to rise through 2030 [1, 3]. Whilst national estimates of the prevalence of DR in Zimbabwe have not been reported, regional estimates vary between 28.4% and 31.1% [4, 5].

DR is the leading cause of blindness in the working‐age population, imposing a significant socio‐economic burden on individuals, their families and wider society; analyses estimate that vision impairment and blindness cause economic productivity losses of $1.71 billion USD in southern sub‐Saharan Africa annually [6]. Early diagnosis of DR is crucial for preventing adverse outcomes such as blindness, as effective management of DM through optimal control of blood glucose and blood pressure can reduce the risk of DR progression once identified [7–10]. Despite the importance of early diagnosis, DR screening programmes are not currently widely available across primary and secondary level facilities in Zimbabwe [11, 12].

Multiple screening methods for DR exist, including (ultrawide) field fundus imaging, optical coherence tomography and the newly emerging field of artificial intelligence (AI)–based devices [13–16]. Digital fundoscopy (DF) is a well established, non‐invasive screening method of capturing images of the retina. DF has proven high sensitivity and specificity, is easy to operate by nonspecialist physicians, and has been shown to be cost‐effective [10, 14, 15, 17].

The primary aims of this study were to assess the feasibility of embedding DF into standard practice to screen for DR at Masvingo Provincial Hospital (MPH), Zimbabwe, which is the referral hospital for the entire Masvingo province; and to assess the prevalence and progression of DR among patients with DM at MPH. The secondary aims of the study were to determine the baseline variables associated with DR and its progression over 1 year. Additionally, exploratory aims included determining the number of participants that experienced DR regression, the proportion of participants who required referral for further tests and the relationship between referral recommendations and DR outcomes.

## 2. Materials and Methods

### 2.1. Ethical Approval

Ethical approval from the Medical Research Council of Zimbabwe was issued under Study Registration Number MRCZ/A/2429 in 2019. The study protocol was also reviewed by the Provincial Medical Director of Masvingo province and the Research Council of Zimbabwe.

### 2.2. Data Collection

Enrolment of participants took place between October 2019 and October 2021. Data from the Noncommunicable Disease (NCD) Clinic at MPH were collated by a research assistant (funded by a non‐governmental organisation) using paper data collection tools and compiled into Microsoft Excel files. There were 290 patients registered at MPH with DM at the time of the study. Of these 290 patients, 208 attended the NCD clinic during the recruitment period and were considered for eligibility in the study. The inclusion criteria for the study were that participants had to be aged 18 or above with a confirmed diagnosis of DM, routinely attending reviews at the MPH NCD Clinic and willing to participate in the study. DM was diagnosed through blood glucose tests, including HbA1c and fasting blood sugar confirmation, following clinical algorithms. Diagnostic tests were carried out by qualified healthcare workers and clinicians. Exclusion criteria included age below 18 years, presence of a condition interfering with the suitability of DR screening and unwillingness to participate in the study. Of the 208 patients assessed, two were excluded due to being blind and two were excluded due to being under 18 years of age. No eligible participants refused to participate. No sample size calculation was performed, given the exploratory nature of the study.

Follow‐up was conducted within the known patient cohort during their routine clinic review visits. Continuity was supported through the use of patient contact details, including phone calls and SMS reminders. Participation in follow‐up remained voluntary and was not subject to coercion.

For patients enrolled in the study, assessment included obtaining a detailed patient history, focusing on DM and its modifiers to assess risk profile, as well as collection of socio‐demographic data. Participants then underwent fundoscopy screening with a Volk digital fundoscopy imaging device (DFID) at baseline and at Year 1 as part of a complete ophthalmic examination, in alignment with programme review schedules in NCD clinics in Zimbabwe and global ophthalmological practice [18, 19].

Fundus photographs were transmitted via a secure, email‐based system using compressed file formats to optimise efficiency. Each image was accompanied by an electronic referral form. The form and images were reviewed by a qualified remote general ophthalmologist. The ophthalmologist assessed the presence of DR and diabetic macular oedema (DME) for each eye, graded the severity of DR and returned their assessment upon completion. The clinician completing the ophthalmic review at MPH then made recommendations for further tests or intervention based on the remote ophthalmologist′s report. An annual fundoscopic exam was recommended for all participants unless the specialist recommended an earlier exam due to abnormalities detected. All transfers adhered to strict confidentiality and data protection protocols.

### 2.3. Demographic and Clinical Variables

To assess the association of baseline variables with the presence of DR at baseline and progression at Year 1, the following demographics and clinical characteristics were collected for each participant: age; biological sex (self‐reported); number of years since DM diagnosis; DM type (based on clinical presentation, age of onset and laboratory investigations such as C‐peptide tests); DM therapy; total cholesterol (normal [< 200 mg/dL], elevated [200–239 mg/dL], high [≥ 240 mg/dL]); triglycerides (normal [< 150 mg/dL], borderline high [150–199 mg/dL], high or very high [≥ 200 mg/dL]); high‐density lipoproteins (HDL) (low [< 40 mg/dL], optimal/normal [≥ 40 mg/dL]); low‐density lipoproteins (LDL) (optimal or near optimal [< 130 mg/dL], borderline high [130–159 mg/dL], high or very high [≥ 160 mg/dL]); creatinine (normal [< 125 *μ*mol/L], elevated [125–199 *μ*mol/L], high [≥ 200 *μ*mol/L]) and haemoglobin A1c (HbA1c) (normal [< 7.0%/< 53 mmol/mol; indicates good diabetic control], elevated [7.0%–7.9%/53–63 mmol/mol; partially controlled diabetes], high [≥ 8.0%/≥ 64 mmol/mol; poorly controlled diabetes]).

To assess the severity of DR and 1‐year DR progression, participants were recorded as having no disease, non‐proliferative diabetic retinopathy (NPDR) (including mild, moderate and severe forms), proliferative diabetic retinopathy (PDR), and/or clinically significant macular oedema (CSME) in each eye at baseline and at Year 1. One‐year DR progression was defined as an increase in severity of DR between baseline and Year 1, based on the International Clinical Disease Severity Scale for DR [20]. If a participant displayed either an increase in the number of eyes diagnosed with the same disease (such as NPDR in one eye at baseline and NPDR in both eyes at Year 1) or detection of newly diagnosed disease (such as no PDR recorded at baseline and PDR recorded in at least one eye at Year 1; participants could experience > 1 type of disease in a given eye), they were considered to have had an increase in severity. Regression was defined as a participant who had any sign of DR at baseline and did not have DR at Year 1.

To assess the feasibility of the use of DF as a new screening method for DR, the time from image capture to the report being returned by the remote ophthalmologist was measured, both at baseline and at Year 1. Any test/intervention referral recommendations made during the baseline visit were recorded for each participant. Referral recommendations were compared with the presence of DR at baseline and at Year 1 and DR progression at Year 1.

### 2.4. Statistical Analyses

All steps in the analyses were conducted by one programmer, with quality control by a second programmer independently. All analyses were undertaken in R (Version 4.1.3 or later) [21]. The dataset was cleaned prior to analysis to ensure all patients met the inclusion criteria.

Overall prevalence and 1‐year progression of DR were estimated, alongside summaries of each variable of interest. Baseline characteristics were also stratified based on baseline HbA1c, DM type and total cholesterol levels. Continuous variables were summarised using the mean and standard deviation (SD), and categorical variables were summarised using counts and percentages. Descriptive summaries were reported using the full analysis approach, where all available data were summarised.

A univariable logistic regression model was fitted to the data to generate unadjusted odds ratios (ORs) for each variable of interest (age, sex, number of years since DM diagnosis, DM type, DM therapy, total cholesterol, triglycerides, HDL, LDL, creatinine and HbA1c) to determine whether there was an association with DR without adjusting for other variables. A multivariable logistic regression model was then fitted to the data to adjust for DM type, number of years with DM, total cholesterol and HbA1c, which were selected as key prognostic variables prior to analysis based on the clinical experience of the authors. The model yielded adjusted ORs. Participants that had any missing characteristic data were excluded from the regression analysis.

## 3. Results and Discussion

### 3.1. Results

The raw dataset included 204 patients. One patient was excluded from the data analyses due to age < 18 years, and one was excluded due to the number of years since their DM diagnosis being larger than their age, leaving 202 eligible participants with baseline and 1‐year follow‐up data included for analysis. Among 202 participants included in the study, 84 (41.6%) had DR at baseline. Baseline characteristics for all eligible participants (*N* = 202) are presented in Table 1, alongside baseline characteristics for participants with DR at baseline (*N* = 84) and participants without DR at baseline (*N* = 118). Baseline characteristics stratified by baseline HbA1c, DM type and total cholesterol levels are presented in Tables S1, S2 and S3.

**Table 1:** Baseline characteristics.

**Characteristic**	**Participants with DR** (**N** = 84)	**Participants without DR** (**N** = 118)	**All participants** (**N** = 202)
Age (years), mean (SD)	57.50 (11.43)	55.99 (13.38)	56.62 (12.60)
Sex, *n* (%)			
Female	69 (82.14)	88 (74.58)	157 (77.72)
Male	15 (17.86)	30 (25.42)	45 (22.28)
Number of years since DM diagnosis, mean (SD)	7.93 (6.50)	6.37 (6.85)	7.02 (6.74)
DM type, *n* (%)			
Type 1	7 (8.33)	10 (8.47)	17 (8.42)
Type 2	77 (91.67)	108 (91.53)	185 (91.58)
DM therapy, *n* (%)			
Oral medications only	72 (85.71)	104 (88.14)	176 (87.13)
Insulin only	11 (13.10)	12 (10.17)	23 (11.39)
Oral medications and insulin	1 (1.19)	2 (1.69)	3 (1.49)
NPDR, *n* (%)			
None	14 (16.67)	118 (100.00)	132 (65.35)
One eye	15 (17.86)	NA	15 (7.43)
Both eyes	55 (65.48)	NA	55 (27.23)
PDR, *n* (%)			
None	55 (65.48)	118 (100.00)	173 (85.64)
One eye	2 (2.38)	NA	2 (0.99)
Both eyes	27 (32.14)	NA	27 (13.37)
CSME, *n* (%)			
None	24 (28.57)	118 (100.00)	142 (70.30)
One eye	17 (20.24)	NA	17 (8.42)
Both eyes	43 (51.19)	NA	43 (21.29)
Total cholesterol (mg/dL), mean (SD)	188.29 (49.48)	185.52 (40.48)	186.67 (44.35)
Triglycerides (mg/dL), mean (SD)	139.33 (68.31)	160.81 (91.26)	151.88 (82.99)
HDL (mg/dL), mean (SD)^a^	56.72 (18.03)	54.64 (15.56)	55.50 (16.61)
LDL (mg/dL), mean (SD)^b^	106.60 (39.70)	103.12 (37.60)	104.55 (38.42)
Creatinine (*μ*mol/L), mean (SD)	69.69 (18.40)	70.38 (31.99)	70.09 (27.12)
HbA1c			
%, mean (SD)	7.67 (2.51)	7.13 (3.06)	7.35 (2.85)
mmol/mol, mean	60	54	57
1‐year DR progression, *n* (%)			
No	73 (86.90)	110 (93.22)	183 (90.59)
Yes	11 (13.10)	8 (6.78)	19 (9.41)

Abbreviations: %, percent; *μ*mol, micromole; mg, milligram; CSME, clinically significant macular oedema; dL, decilitre; DM, diabetes mellitus; DR, diabetic retinopathy; HbA1c, haemoglobin A1c; HDL, high‐density lipoprotein; L, litre; LDL, low‐density lipoprotein; *N*, total number of patients; *n*, number of patients in subgroup; NA, not applicable; NPDR, non‐proliferative diabetic retinopathy; PDR, proliferative diabetic retinopathy; SD, standard deviation.

^a^One patient had missing data for HDL at baseline.

^b^Three patients had missing data for LDL at baseline.

**Figure 1:**
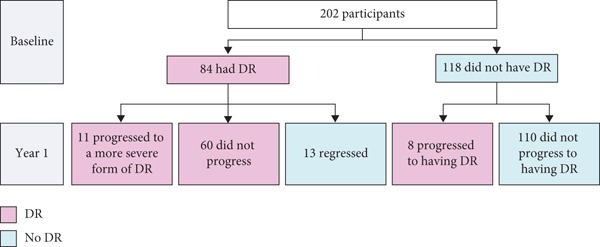
Number of participants with DR/DR progression at baseline and Year 1. *Note:* Raw dataset included 204 patients. One patient was excluded from the data analyses due to age < 18 years, and one was excluded due to the number of years since their DM diagnosis being larger than their age. Abbreviations: DR, diabetic retinopathy.

Among the 118 participants without DR at baseline, eight had DR at Year 1, which translated to an observed incidence of 6.8% during 1 year of follow‐up, equivalent to a rate of 68 cases per 1000 person years (Figure 1). In contrast, 13 participants (15.5%) who had DR at baseline regressed at Year 1. During the 1‐year follow‐up, a total of 19 participants (9.4%) experienced progression of DR, including eight (6.8%) who did not have DR at baseline and 11 (13.1%) who did.

Figure 2 presents the unadjusted and adjusted associations of variables with DR at baseline, as identified through ORs. Participants with elevated HbA1c (7.0%–7.9%/53–63 mmol/mol) had significantly higher adjusted odds of having DR compared with participants with normal HbA1c (< 7.0%/< 53 mmol/mol) (adjusted OR: 3.43; 95% confidence interval [CI]: 1.18, 10.83; *p* = 0.03). Participants with high HbA1c (≥ 8.0%/≥ 64 mmol/mol) had 1.56 higher adjusted odds of having DR than those with normal HbA1c (95% CI: 0.83; 2.93); however, this was not statistically significant (*p* = 0.17).

Figure 2(a) Unadjusted and (b) adjusted association of baseline variables with DR at baseline. ORs for patients with elevated or high creatinine could not be estimated due to the small number of patients observed in these groups. Adjusted associations were adjusted for DM type, number of years since DM diagnosis, total cholesterol and HbA1c. ∗ = *p* < 0.05. Abbreviations: %, percent; *μ*mol, micromole; CI, confidence interval; dL, decilitre; DM, diabetes mellitus; DR, diabetic retinopathy; HbA1c, haemoglobin A1c; HDL, high‐density lipoprotein; L, litre; LDL, low‐density lipoprotein; mg, milligram; OR, odds ratio. *Note:* The lab values were categorised as follows: total cholesterol (normal, < 200 mg/dL; elevated, 200–239 mg/dL; high, ≥ 240 mg/dL), triglycerides (normal, < 150 mg/dL; borderline high, 150–199 mg/dL; high or very high, ≥ 200 mg/dL), HDL (low, < 40 mg/dL; optimal/normal, ≥ 40 mg/dL), LDL (optimal or near optimal, < 130 mg/dL; borderline high, 130–159 mg/dL; high or very high, ≥ 160 mg/dL), creatinine (normal, < 125 *μ*mol/L; elevated, 125–199 *μ*mol/L; high, ≥ 200 *μ*mol/L) and HbA1c (normal, < 7.0%/< 53 mmol/mol; elevated, 7.0%–7.9%/53–63 mmol/mol; high, ≥ 8.0%/≥ 64 mmol/mol). ORs for patients with elevated or high creatinine could not be estimated due to the small number of patients observed in these groups. Adjusted associations were adjusted for DM type, number of years since DM diagnosis, total cholesterol and HbA1c. ∗ = *p* < 0.05. Abbreviations: %, percent; *μ*mol, micromole; CI, confidence interval; dL, decilitre; DM, diabetes mellitus; DR, diabetes retinopathy; HbA1c, haemoglobin A1c; HDL, high‐density lipoprotein; L, litre; LDL, low‐density lipoprotein; mg, milligram; OR, odds ratio.

**Figure:**
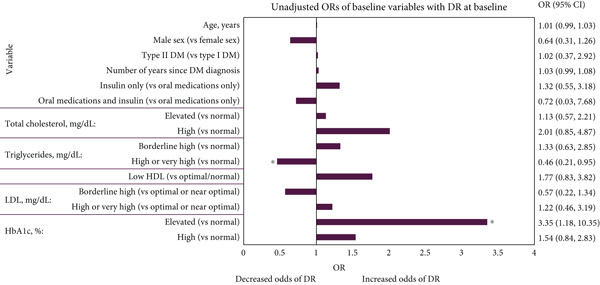
(a)

**Figure:**
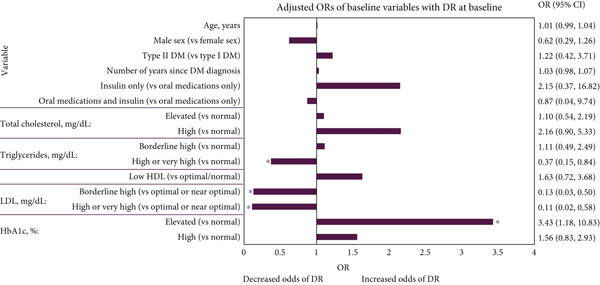
(b)

Participants had significantly lower adjusted odds of having DR if they had borderline high (130–159 mg/dL; adjusted OR: 0.13; 95% CI: 0.03, 0.50; *p* = 0.01) or high/very high LDL (≥ 160 mg/dL; adjusted OR: 0.11; 95% CI: 0.02, 0.58; *p* = 0.01) compared with participants with optimal or near optimal LDL (< 130 mg/dL) (Figure 2). Participants with high or very high triglycerides (≥ 200 mg/dL) had significantly lower adjusted odds of having DR than participants with normal triglycerides (< 150 mg/dL; adjusted OR: 0.37; 95% CI: 0.15, 0.84; *p* = 0.02) (Figure 2). No other variables were associated with significantly different adjusted odds of having DR at baseline. Low numbers of participants with elevated/high creatinine meant association estimates could not be made for this variable.

As the sample was skewed towards a considerably higher percentage of female than male participants (77.7% female, 22.3% male), the multivariable logistic regression analysis of variables associated with the presence of DR at baseline was rerun to account for this. This did not significantly impact any of the results; the same associations, or lack thereof, were found for each variable (Table S4).

**Figure 3:**
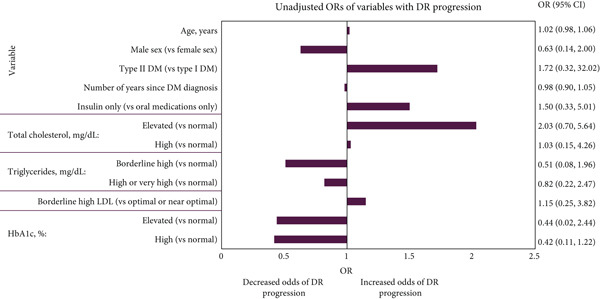
Unadjusted association of baseline variables with DR progression. Adjusted logistic regression was not performed due to the small number of patients with DR progression. ORs for patients receiving both oral medications and insulin, patients with low HDL, high or very high LDL or elevated or high creatinine could not be estimated due to the small number of patients observed in these groups. Abbreviations: %, percent; *μ*mol, micromole; CI, confidence interval; dL, decilitre; DM, diabetes mellitus; DR, diabetic retinopathy; HbA1c, haemoglobin A1c; HDL, high‐density lipoprotein; L, litre; LDL, low‐density lipoprotein; mg, milligram; OR, odds ratio. *Notes:* The lab values were categorised as follows: total cholesterol (normal, < 200 mg/dL; elevated, 200–239 mg/dL; high, ≥ 240 mg/dL), triglycerides (normal, < 150 mg/dL; borderline high, 150–199 mg/dL; high or very high, ≥ 200 mg/dL), LDL (optimal or near optimal, < 130 mg/dL; borderline high, 130–159 mg/dL; high or very high, ≥ 160 mg/dL), and HbA1c (normal, < 7.0%/< 53 mmol/mol; elevated, 7.0%–7.9%/53–63 mmol/mol; high, ≥ 8.0%/≥ 64 mmol/mol). ORs with CIs for patients receiving both oral medications and insulin, patients with low HDL, high or very high LDL or elevated or high creatinine could not be estimated due to the small number of patients observed in these groups. Abbreviations: %, percent; *μ*mol, micromole; CI, confidence interval; dL, decilitre; DM, diabetes mellitus; DR, diabetes retinopathy; HbA1c, haemoglobin A1c; HDL, high‐density lipoprotein; L, litre; LDL, low‐density lipoprotein; mg, milligram; OR, odds ratio.

Figure 3 presents the unadjusted associations of baseline variables with DR progression. No variables were found to be significantly associated with DR progression, and some association estimates were incalculable due to low numbers of participants. Adjusted logistic regression for DR progression was not performed due to the small number of participants with DR progression.

The mean time between transfer of images captured using the DFID and return of the completed report was 36.16 days at baseline and 18.89 days at Year 1. Of the 202 participants who underwent DF screening at baseline, 136 were recommended for further tests or intervention, including 63 of the 84 participants with DR at baseline. Among these 63 participants, 10 experienced disease progression by Year 1, whilst 53 did not progress. In contrast, of the 21 participants with DR at baseline who were not recommended for further tests or intervention, only one progressed by Year 1. Additionally, of the eight participants who did not have DR at baseline but developed DR by Year 1, six had been recommended for further tests or intervention at baseline.

### 3.2. Discussion

The estimated prevalence of DR among patients with DM across Africa is 35.9%, whilst regional estimates across Zimbabwe range from 28.4% to 31.1% [1, 4, 5]. We conducted a single‐site study at MPH, the referral hospital for the entire province of Masvingo, which receives patients from both the urban and surrounding districts, to assess the prevalence and progression of DR in the province. Our results suggest that the prevalence of DR is higher in Masvingo than in other regions of Zimbabwe, and across Africa more widely, with an estimated prevalence of 41.6% among patients with DM found in our study. This high rate of DR is in line with evidence demonstrating that health outcomes in Masvingo are poor in comparison to other provinces in Zimbabwe, with a high occurrence of conditions such as hypertension, HIV and AIDS [22, 23]. This is further exacerbated by deprivation, droughts and underdevelopment prevalent in the province [24]. Among participants without DR at baseline, the incidence of DR was 6.8% during 1 year of follow‐up (a rate of 68 cases per 1000 person years). A 2013 study estimated that there were 601,000 people with DM in Zimbabwe at the time. By applying the incidence rate and proportion of patients with DR observed in Masvingo province, as determined in the present study, to national population data, it is estimated that there could be up to approximately 24,000 new cases of DR each year in Zimbabwe [25]. Furthermore, the burden of DR has been reported to be increasing in Zimbabwe and across Africa; the same study predicted that by 2035 there will be 1.256 million people with DM in Zimbabwe [3, 25]. The number of new cases of DR per year in Zimbabwe may therefore increase to ~50,000 by 2036. These results demonstrate the scale of the problem and highlight the need for increased availability of DR screening and access to treatment options to reduce the prevalence and adverse outcomes of DR.

Age and number of years since DM diagnosis were not significantly associated with the odds of DR at baseline. These findings contradict previous literature suggesting that a higher number of years with DM is associated with the presence of DR [26–28]. One potential explanation may be that the odds of DR are more closely linked to DM control, proxied by HbA1c levels, than the number of years with DM, as DR is less likely to occur among patients with controlled DM [29]. Additionally, the difference between our results and those of previous studies may be due to differences in how age and the number of years since DM diagnosis were analysed. Our study analysed these as continuous variables to allow us to understand the incremental odds per year. Whilst small for every passing year, the increase may potentially be more pronounced in 5‐ or 10‐year discrete categories, as used in other studies [26, 27].

The unadjusted and adjusted odds of having DR at baseline were significantly higher for participants with elevated HbA1c (7.0%–7.9%/53–63 mmol/mol) and numerically higher for participants with high HbA1c (≥ 8.0%/≥ 64 mmol/mol) compared with normal HbA1c. These data suggest that patients with poor glycaemic control, as indicated by elevated HbA1c levels, have higher odds of having DR, which is in line with previous studies [30]. It is likely that participants with the highest HbA1c levels received more clinical attention relating to their DM than participants with normal HbA1c levels, as recommended by clinical guidelines [9, 29]. This may explain why the higher odds of DR among these participants, compared with participants with normal HbA1c levels, did not reach statistical significance. These findings highlight the importance of effective management of DM to reduce the likelihood of developing DR and suggest that patients with poorly controlled DM, as indicated by elevated HbA1c levels, could be prioritised for DR screening programmes. In our study, patients with elevated HbA1c levels did not have higher odds of DR progression, contradicting existing literature [29]. However, the small sample size of our study limits the conclusions that can be made regarding DR progression and poor glycaemic control in Masvingo province, warranting further investigation.

We also found that adjusted odds of having DR at baseline were significantly lower for participants with high or very high triglycerides or borderline high, high or very high LDL, compared with normal, optimal or near optimal levels. Previous studies investigating these relationships have reported mixed results on whether these variables are significantly associated with odds of DR, suggesting further research is warranted [29, 31–34].

The time between capture of images using the DF device and the return of the completed report from the remote ophthalmologist was 36.16 days at baseline and 18.89 days at Year 1. The differences in report turnaround time between baseline and Year 1 are likely due to learning and increased familiarity with the screening pathway. The mean turnaround time aligns with global guidelines and the turnaround reported across other real‐world studies, suggesting that using DF for DR screening is feasible to embed into standard practice in this setting [35–38]. The time‐limiting factor is the time required for the ophthalmologist to review each image, and alternative technologies, such as AI‐based DFIDs, could be tested in this setting to facilitate scale up beyond the Masvingo province [16].

The increasing burden of DR in Africa suggests that effective DR screening is urgently needed, especially given that many adverse DR outcomes such as blindness are preventable if effective management strategies are implemented early [3, 8]. This study suggests that large‐scale DR screening programmes could be implemented in referral hospitals such as MPH and would allow for early detection and intervention, reducing the individual and society‐level consequences of DR. However, further analyses are required to review the technicalities, sensitivity and specificity of DF as a screening method, as well as its acceptability to healthcare workers in Zimbabwe. It should be noted that a full‐time research assistant conducted the screening for this study, which does not reflect how the system is currently administered by Zimbabwe′s MoHCC. Additional resources for facilitating screening would therefore need to be considered by the MoHCC for effective implementation.

This study has several notable strengths. Firstly, the wide range of variables included in the dataset allowed for thorough investigation of the association of demographic and clinical characteristics with DR. The collection of these clinically relevant variables allowed ORs for presence of DR at baseline to be adjusted for DM type, number of years since DM diagnosis, total cholesterol and HbA1c. Secondly, the decisions made regarding the management of confounders were based on clinical input, rather than statistical observations, allowing for more clinically relevant conclusions to be drawn. Finally, minimal missing data at baseline meant that most records were of complete cases, meaning almost all participants could be included in the regression analysis.

It should be noted that there was a high proportion of female participants in our study population (77.7%) compared with males (22.3%). Potential reasons for this may include the demographics of the patients who attend MPH. Health‐seeking behaviours are known to be less common among men, particularly in Africa, and clinicians based at the hospital have anecdotally reported a higher frequency of female than male patients [39, 40]. Our analysis showed that the skew towards females in our sample did not impact which variables were associated with the presence of DR at baseline. However, previous literature suggests that DR is more common in men than women overall, raising the possibility that our results may be an underestimate of the true magnitude of DR in Masvingo. This would be further exacerbated by undiagnosed DM cases; it is thought that in Africa, 53.6% of DM cases are not diagnosed [41–43]. This reinforces the urgent need for enhanced healthcare resources for DR screening and the importance of implementing strategies to ensure its accessibility for all individuals at risk of DR.

Our study provides valuable early insights—particularly as the 1‐year follow‐up period aligns with programme review schedules in Zimbabwean NCD clinics and global ophthalmological practice, which recommends yearly retinal screening for individuals with DM, including those without sight‐threatening disease [18, 19]. Yet, a longer follow‐up would have been preferable to fully capture the natural history of DR progression, especially in patients at early stages or with slow disease progression [18]. The study′s limited duration, together with the small number of cases demonstrating disease progression, restricted the assessment of unadjusted associations between certain variables and precluded the calculation of adjusted associations, thereby limiting the strength of the conclusions that can be drawn. This could be investigated further through future research with more participants and a longer follow‐up time, which would increase the robustness and reliability of the odds of progression.

It should be considered that recruiting participants from an NCD clinic may introduce selection bias by excluding undiagnosed or nonattending individuals with diabetes. However, this population is the most likely to be reached through a screening programme and is thus especially pertinent to service delivery planning. Validation of screening outcomes through dual review would also have increased the study′s reliability; however, this was not feasible due to resource constraints. Nonetheless, all cases referred for surgery following a DR diagnosis in the DF referral pathway were independently confirmed by an ophthalmologist, supporting the reliability of remote image interpretation in this context. Other potential limitations of our dataset include that it may not accurately reflect how long a participant has been living with DM, if there had been a delay in diagnosis. Exposures and effect modifiers were also not explicitly defined in this study. Finally, due to a lack of available evidence on the clinical significance of the ORs, the study only considered the statistical significance of the association of variables with DR and DR progression.

## 4. Conclusions

Our study shows that the magnitude of DR is high in Masvingo, Zimbabwe, providing compelling evidence of the need for increased healthcare resources for DR screening in this setting, and clearly demonstrates the feasibility of embedding DF into standard practice for this purpose. Our results suggest that patients with poorly managed DM, as indicated by elevated HbA1c, could be targeted for DR screening programmes. We strongly recommend that these results are taken into consideration in future decision‐making regarding screening and healthcare provision in Zimbabwe. Additionally, further investigation is needed on the variables associated with DR progression to support future decision‐making on the implementation of effective screening programmes.

## Disclosure

All authors edited, reviewed and approved the final version of the manuscript.

## Conflicts of Interest

M.K. is an employee of MOHCC. K.M. is an employee of SolidarMed. M.M. is employee of Costello Medical. A.T.M.M. is an employee of SolidarMed. A.N.M. is an employee of SolidarMed. He pursued and completed a Master′s thesis on the prevalence of diabetic retinopathy, assessing progression and regression of DR after Year 1 review. A.N. is an employee of Costello Medical. L.E.R. is an employee of SolidarMed.

## Author Contributions

A.T.M.M. was involved in proposal review, implementation of the study, testing of lipid profile and HbA1c, analysis of creatinine results, DR screening of patients, sending of patient referrals for diagnosis, data collection, documentation, preliminary analysis and interpretation of data. M.K. conducted patient fundoscopy review, diagnosed and graded DR and interpreted data. A.N.M. was involved in study conception, design and protocol writing, was the study principal investigator, facilitated study approval and research council authorisation, conducted data analysis, and was involved in study review, implementation consultation and interpretation of data. K.M. was responsible for study project teams and implementation oversight, statistical data review, preliminary manuscript data review and the project reports review. A.N. and M.M. advised on the data analysis approach and conducted data analysis. L.E.R. advised on interpretation of data. Medical writing support was provided by Costello Medical, under the direction of all authors.

## Funding

This study was supported by SolidarMed, Switzerland.

## Supporting information


**Supporting Information** Additional supporting information can be found online in the Supporting Information section. Table S1: Baseline characteristics stratified by HbA1c levels. Table S2: Baseline characteristics stratified by DM type. Table S3: Baseline characteristics stratified by cholesterol levels. Table S4: Adjusted association of baseline variables with DR at baseline, including adjustment for sex.

## Data Availability

The datasets generated and/or analysed during the current study are kept on SolidarMed repository and are not publicly available due to provisions within our Memorandum of Understanding (MoU), Article 15 on ownership and use of data, with the Ministry of Health and Child Care (MoHCC). The MoU specifies that data produced during the duration of each 5‐year MoU agreement phase and the programmes initiated thereunder shall be owned by the Government of Zimbabwe (GoZ). As such, persons involved in the implementation of the programmes established through the MoU who wish to use the said data shall seek permission from the GoZ. Such permission shall not be unfairly withheld, which makes the data potentially available from the corresponding author on reasonable request.
