# Supplementary material for: Diabetic Retinopathy Prevalence and Incidence in Zimbabwe: The Feasibility of Digital Fundoscopy Screening
Source: J Diabetes Res. 2026 Jan 26;2026:8048762. doi: 10.1155/jdr/8048762 (PMC12833612; doi:10.1155/jdr/8048762)
Supplement: Supplementary file 1 — Supporting Information Additional supporting information can be found online in the Supporting Information section. Table S1: Baseline characteristics stratified by HbA1c levels. Table S2: Baseline characteristics stratified by DM type. Table S3: Baseline characteristics stratified by cholesterol levels. Table S4: Adjusted association of baseline variables with DR at baseline, including adjustment for sex. [file JDR-2026-8048762-s001.docx]

**Supplementary Materials**

Table S1. Baseline characteristics stratified by HbA1c levels

| **Characteristic** | **HbA1c <7.0% (<53 mmol/mol) (N=113)** | **HbA1c 7.0–7.9% (53–63 mmol/mol) (N=17)** | **HbA1c ≥8% (≥64 mmol/mol) (N=72)** |
| --- | --- | --- | --- |
| **Age (years), mean (SD)** | 59.50 (11.10) | 56.24 (10.47) | 52.18 (14.03) |
| **Sex, n (%)** | | | |
| Female | 92 (81.42) | 14 (82.35) | 51 (70.83) |
| Male | 21 (18.58) | 3 (17.65) | 21 (29.17) |
| **DM type, n (%)** | | | |
| Type I | 4 (3.54) | 1 (5.88) | 12 (16.67) |
| Type II | 109 (96.46) | 16 (94.12) | 60 (83.33) |
| **Number of years since DM diagnosis, mean (SD)** | 6.40 (6.14) | 9.53 (6.38) | 7.4 (7.59) |
| **DM therapy, n (%)** | | | |
| Oral medications only | 102 (90.27) | 16 (94.12) | 58 (80.56) |
| Insulin only | 8 (7.08) | 1 (5.88) | 14 (19.44) |
| Oral medications and insulin | 3 (2.65) | 0 (0.00) | 0 (0.00) |
| **NPDR, n (%)** | | | |
| None | 80 (70.80) | 7 (41.18) | 45 (62.50) |
| One eye | 9 (7.96) | 0 (0.00) | 6 (8.33) |
| Both eyes | 24 (21.24) | 10 (58.82) | 21 (29.17) |
| **PDR, n (%)** | | | |
| None | 100 (88.50) | 11 (64.71) | 62 (86.11) |
| One eye | 2 (1.77) | 0 (0.00) | 0 (0.00) |
| Both eyes | 11 (9.73) | 6 (35.29) | 10 (13.89) |
| **CSME, n (%)** | | | |
| None | 84 (74.34) | 9 (52.94) | 49 (68.06) |
| One eye | 13 (11.50) | 0 (0.00) | 4 (5.56) |
| Both eyes | 16 (14.16) | 8 (47.06) | 19 (26.39) |
| **Total cholesterol (mg/dL), mean (SD)** | 187.16 (44.89) | 183.65 (40.02) | 186.61 (45.00) |
| **Triglycerides (mg/dL), mean (SD)** | 153.58 (82.81) | 146.18 (76.40) | 150.56 (85.74) |
| **HDL (mg/dL), mean (SD)*** | 56.05 (14.73) | 54.53 (22.43) | 54.86 (17.99) |
| **LDL (mg/dL), mean (SD)**^†^ | 104.42 (35.62) | 104.12 (38.69) | 104.85 (42.87) |
| **Creatinine (µmol/L), mean (SD)** | 70.37 (31.44) | 70.71 (18.95) | 69.5 (21.00) |
| **HbA1c** | | | |
| %, mean (SD) | 5.28 (0.96) | 7.35 (0.29) | 10.61 (1.97) |
| mmol/mol, mean | 34 | 57 | 92 |
| **1-year DR progression, n (%)** | | | |
| No | 99 (87.61) | 16 (94.12) | 68 (94.44) |
| Yes | 14 (12.39) | 1 (5.88) | 4 (5.56) |

Footnotes: * One patient had missing data for HDL at baseline; ^†^ Three patients had missing data for LDL at baseline.
Abbreviations: %, percent; µmol, micromole; CSME, clinically significant macular oedema; dL, decilitre; DM, diabetes mellitus; DR, diabetic retinopathy; HbA1c, haemoglobin A1c; HDL, high-density lipoprotein; L, litre; LDL, low-density lipoprotein; mg, milligram; N, total number of patient; n, number of patients in subgroup; NA, not applicable; NPDR, non-proliferative diabetic retinopathy; PDR, proliferative diabetic retinopathy; SD, standard deviation.

Table S2. Baseline characteristics stratified by DM type

| **Characteristic** | **DM type I (N=17)** | **DM type II (N=185)** |
| --- | --- | --- |
| **Presence of DR, n (%)** | | |
| No | 10 (58.82) | 108 (58.38) |
| Yes | 7 (41.18) | 77 (41.62) |
| **Age (years), mean (SD)** | 42.41 (15.02) | 57.92 (11.55) |
| **Sex, n (%)** | | |
| Female | 11 (64.71) | 146 (78.92) |
| Male | 6 (35.29) | 39 (21.08) |
| **Number of years since DM diagnosis, mean (SD)** | 7.24 (5.90) | 7.00 (6.82) |
| **DM therapy, n (%)** | | |
| Oral medications only | 0 (0.00) | 176 (95.14) |
| Insulin only | 17 (100.00) | 6 (3.24) |
| Oral medications and insulin | 0 (0.00) | 3 (1.62) |
| **NPDR, n (%)** | | |
| None | 12 (70.59) | 120 (64.86) |
| One eye | 1 (5.88) | 14 (7.57) |
| Both eyes | 4 (23.53) | 51 (27.57) |
| **PDR, n (%)** | | |
| None | 13 (76.47) | 160 (86.49) |
| One eye | 0 (0.00) | 2 (1.08) |
| Both eyes | 4 (23.53) | 23 (12.43) |
| **CSME, n (%)** | | |
| None | 11 (64.71) | 131 (70.81) |
| One eye | 1 (5.88) | 16 (8.65) |
| Both eyes | 5 (29.41) | 38 (20.54) |
| **Total cholesterol (mg/dL), mean (SD)** | 187.47 (51.15) | 186.59 (43.83) |
| **Triglycerides (mg/dL), mean (SD)** | 125.76 (54.93) | 154.28 (84.81) |
| **HDL (mg/dL), mean (SD)*** | 54.41 (14.50) | 55.6 (16.82) |
| **LDL (mg/dL), mean (SD)**^†^ | 106.29 (47.79) | 104.39 (37.58) |
| **Creatinine (µmol/L), mean (SD)** | 59.09 (15.46) | 71.1 (27.76) |
| **HbA1c** | | |
| %, mean (SD) | 9.81 (3.54) | 7.13 (2.68) |
| mmol/mol, mean | 84 | 54 |
| **1-year DR progression, n (%)** | | |
| No | 16 (94.12) | 167 (90.27) |
| Yes | 1 (5.88) | 18 (9.73) |

Footnotes: * One patient had missing data for HDL at baseline; ^†^ Three patients had missing data for LDL at baseline.
Abbreviations: %, percent; µmol, micromole; CSME, clinically significant macular oedema; dL, decilitre; DM, diabetes mellitus; DR, diabetic retinopathy; HbA1c, haemoglobin A1c; HDL, high-density lipoprotein; L, litre; LDL, low-density lipoprotein; mg, milligram; N, total number of patient; n, number of patients in subgroup; NA, not applicable; NPDR, non-proliferative diabetic retinopathy; PDR, proliferative diabetic retinopathy; SD, standard deviation.

Table S3. Baseline characteristics stratified by cholesterol levels

| **Characteristic** | **Cholesterol <200 mg/dL (N=129)** | **Cholesterol 200–239 mg/dL (N=48)** | **Cholesterol ≥240 mg/dL (N=25)** |
| --- | --- | --- | --- |
| **Presence of DR, n (%)** | | | |
| No | 79 (61.24) | 28 (58.33) | 11 (44.00) |
| Yes | 50 (38.76) | 20 (41.66) | 14 (56.00) |
| **Age (years), mean (SD)** | 57.17 (12.41) | 55.1 (13.38) | 56.68 (12.28) |
| **Sex, n (%)** | | | |
| Female | 100 (77.52) | 35 (72.92) | 22 (88.00) |
| Male | 29 (22.48) | 13 (27.08) | 3 (12.00) |
| **DM type, n (%)** | | | |
| Type I | 11 (8.53) | 3 (6.25) | 3 (12.00) |
| Type II | 118 (91.47) | 45 (93.75) | 22 (88.00) |
| **Number of years since DM diagnosis, mean (SD)** | 7.16 (7.31) | 5.94 (5.26) | 8.36 (6.05) |
| **DM therapy, n (%)** | | | |
| Oral medications only | 113 (87.6) | 45 (93.75) | 18 (72.00) |
| Insulin only | 14 (10.85) | 3 (6.25) | 6 (24.00) |
| Oral medications and insulin | 2 (1.55) | 0 (0.00) | 1 (4.00) |
| **NPDR, n (%)** | | | |
| None | 81 (62.79) | 35 (72.92) | 16 (64.00) |
| One eye | 12 (9.30) | 2 (4.17) | 1 (4.00) |
| Both eyes | 36 (27.91) | 11 (22.92) | 8 (32.00) |
| **PDR, n (%)** | | | |
| None | 111 (86.05) | 41 (85.42) | 21 (84.00) |
| One eye | 2 (1.55) | 0 (0.00) | 0 (0.00) |
| Both eyes | 16 (12.40) | 7 (14.58) | 4 (16.00) |
| **CSME, n (%)** | | | |
| None | 97 (75.19) | 31 (64.58) | 14 (56.00) |
| One eye | 8 (6.20) | 5 (10.42) | 4 (16.00) |
| Both eyes | 24 (18.60) | 12 (25.00) | 7 (28.00) |
| **Total cholesterol (mg/dL), mean (SD)** | 160.69 (27.67) | 216.71 (12.99) | 263.04 (23.49) |
| **Triglycerides (mg/dL), mean (SD)** | 133.76 (68.71) | 172.69 (90.54) | 205.44 (104.05) |
| **HDL (mg/dL), mean (SD)*** | 53.82 (16.65) | 59.00 (16.44) | 57.5 (16.09) |
| **LDL (mg/dL), mean (SD)**^†^ | 85.19 (27.33) | 126.59 (21.78) | 166.38 (23.63) |
| **Creatinine (µmol/L), mean (SD)** | 69.23 (19.22) | 74.76 (44.09) | 65.56 (17.71) |
| **HbA1c** | | | |
| %, mean (SD) | 7.29 (2.84) | 7.27 (2.57) | 7.84 (3.41) |
| mmol/mol, mean | 56 | 56 | 62 |
| **1-year DR progression, n (%)** | | | |
| No | 119 (92.25) | 41 (85.42) | 23 (92.00) |
| Yes | 10 (7.75) | 7 (14.58) | 2 (8.00) |

Footnotes: * One patient had missing data for HDL at baseline; ^†^ Three patients had missing data for LDL at baseline.
Abbreviations: %, percent; µmol, micromole; CSME, clinically significant macular oedema; dL, decilitre; DM, diabetes mellitus; DR, diabetic retinopathy; HbA1c, haemoglobin A1c; HDL, high-density lipoprotein; L, litre; LDL, low-density lipoprotein; mg, milligram; N, total number of patient; n, number of patients in subgroup; NA, not applicable; NPDR, non-proliferative diabetic retinopathy; PDR, proliferative diabetic retinopathy; SD, standard deviation.

Table S4. Adjusted association of baseline variables with DR at baseline, including adjustment for sex

| **Variable** | **Odds ratio (95% CIs)** | **P value** |
| --- | --- | --- |
| Number of years since DM diagnosis | 1.03 (0.99–1.08) | 0.20 |
| Type II DM (vs type I DM) | 1.16 (0.40–3.56) | 0.79 |
| Elevated (vs normal) total cholesterol (mg/dL) | 1.13 (0.55–2.28) | 0.73 |
| High (vs normal) total cholesterol (mg/dL) | 2.05 (0.85–5.09) | 0.11 |
| Elevated (vs normal) HbA1c (%) | 3.37 (1.15–10.69) | 0.03 |
| High (vs normal) HbA1c (%) | 1.63 (0.86–3.09) | 0.13 |
| Male sex (vs female sex) | 0.62 (0.29–1.26) | 0.19 |
| Age (years) | 1.01 (0.99–1.04) | 0.26 |
| Insulin only (vs oral medications only) | 2.13 (0.36–16.68) | 0.42 |
| Oral medications and insulin (vs oral medications only) | 0.82 (0.04–9.15) | 0.87 |
| Borderline high (vs normal) triglycerides (mg/dL) | 1.08 (0.48–2.43) | 0.85 |
| High or very high (vs normal) triglycerides (mg/dL) | 0.37 (0.15–0.84) | 0.02 |
| Low (vs optimal/normal) HDL (mg/dL) | 1.80 (0.79–4.15) | 0.16 |
| Borderline high (vs optimal or near optimal) LDL (mg/dL) | 0.14 (0.03–0.53) | 0.007 |
| High or very high (vs optimal or near optimal) LDL (mg/dL) | 0.12 (0.02–0.63) | 0.02 |

Footnotes: The lab values were categorised as follows: total cholesterol (normal, <200 mg/dL; elevated, 200–239 mg/dL; high, ≥240 mg/dL), triglycerides (normal, <150 mg/dL; borderline high, 150–199 mg/dL; high or very high, ≥200 mg/dL), HDL (low, <40 mg/dL; optimal/normal, ≥40 mg/dL), LDL (optimal or near optimal, <130 mg/dL; borderline high, 130–159 mg/dL; high or very high, ≥160 mg/dL), and HbA1c (normal, <7.0%/<53 mmol/mol; elevated, 7.0–7.9%/53–63 mmol/mol; high, ≥8.0%/≥64 mmol/mol). ORs with CIs for patients with elevated or high creatinine could not be estimated due to the small number of patients observed in these groups. Adjusted associations were adjusted for DM type, number of years since DM diagnosis, total cholesterol, HbA1c and sex.

Abbreviations: %, percent; µmol, micromole; CI, confidence interval; dL, decilitre; DM, diabetes mellitus; DR, diabetes retinopathy; HbA1c, haemoglobin A1c; HDL, high-density lipoprotein; L, litre; LDL, low-density lipoprotein; mg, milligram; OR, odds ratio.
